# Gut Microbiota Profile and Functional Gastrointestinal Disorders in Infants: A Longitudinal Study

**DOI:** 10.3390/nu17040701

**Published:** 2025-02-16

**Authors:** Alexandru Cosmin Pantazi, Cristina Maria Mihai, Ancuta Lupu, Adriana Luminita Balasa, Tatiana Chisnoiu, Larisia Mihai, Corina Elena Frecus, Adina Ungureanu, Sergiu Ioachim Chirila, Wassan Nori, Vasile Valeriu Lupu, Ramona Mihaela Stoicescu, Ginel Baciu, Simona Claudia Cambrea

**Affiliations:** 1 Faculty of Medicine, “Ovidius” University of Constanta, 900470 Constanta, Romania; pantazi.cosmin@365.univ-ovidius.ro (A.C.P.); cristina_mihai@365.univ-ovidius.ro (C.M.M.); adriana.balasa@365.univ-ovidius.ro (A.L.B.); larisia_mihai@yahoo.com (L.M.); frecuscorina@yahoo.com (C.E.F.); adina.ungureanu@365.univ-ovidius.ro (A.U.); sergiu.chirila@univ-ovidius.ro (S.I.C.); claudia.cambrea@365.univ-ovidius.ro (S.C.C.); 2 Department of Pediatrics, Clinical Emergency Hospital of Constanta, 900591 Constanta, Romania; 3 Department of Pediatrics, “Grigore T. Popa” University of Medicine and Pharmacy, 700115 Iasi, Romania; vasile.lupu@umfiasi.ro; 4 College of Medicine, Mustansiriyah University, Baghdad 10052, Iraq; dr.wassan76@uomustansiriyah.edu.iq; 5 Department of Microbiology and Immunology, Faculty of Pharmacy, “Ovidius” University of Constanta, 900470 Constanta, Romania; ramona.stoicescu@univ-ovidius.ro; 6 Department of Pediatrics, Faculty of Medicine and Pharmacy, “Dunarea de Jos” University of Galati, 800008 Galati, Romania; ginelbaciu@yahoo.com

**Keywords:** functional gastrointestinal disorders, infants, intestinal microbiota, acidifying flora

## Abstract

Background/Objectives: The gut microbiota is involved in modulating gastrointestinal function and consequently contributes to the manifestation of functional gastrointestinal disorders (FGIDs). The aim of the study was to analyze the composition of the gut microbiota in infants with functional gastrointestinal disorders (infantile colic, functional constipation, gastroesophageal reflux, functional diarrhea) according to age, environmental factors, and clinical manifestations. Methods: The study involved the clinical and laboratory examination of 134 infants divided into two groups: group I (*n* = 82) with FGIDs according to Rome IV criteria, divided into four subgroups (infantile colic, functional constipation, gastroesophageal reflux, and functional diarrhea), and group II (*n* = 52) without FGIDs. To assess the composition of intestinal microbiota, a bacteriological analysis of fecal samples was performed. Results: Infants with functional gastrointestinal disorders presented an imbalance of intestinal microflora, which was characterized by a significant decrease in the main representatives of acidifying flora represented by *Lactobacillus*, *Bifidobacterium*, and *Enterococcus* and high abundance of proteolytic microorganisms from the Enterobacteriaceae family such as *Klebsiella* species and *Escherichia coli*. In infants born by cesarean section or artificially fed, the incidence of functional gastrointestinal disorders and intestinal dysbiosis was significantly higher. Conclusions: The imbalance of acidifying and proteolytic microbial composition in the gut could be the key to the occurrence of functional gastrointestinal disorders in the first year of life.

## 1. Introduction

The gut microbiota plays a crucial role in various physiological processes, including digestion, immune system modulation, and protection against pathogens [1].

Its establishment in early life, particularly during infancy, is a critical period that may influence long-term health outcomes. Recent advances have highlighted the potential link between gut microbiota composition and functional gastrointestinal disorders (FGIDs) in children and infants. Understanding this relationship is essential for identifying potential microbial biomarkers and developing targeted interventions for FGIDs.

FGIDs, such as gastroesophageal reflux, functional constipation, and functional diarrhea, are diagnosed based on clinical symptoms when no detectable structural or biochemical irregularities are present [2]. Although the exact etiopathogenesis of FGIDs remains unclear, a growing body of evidence suggests that the gut microbiota may play a role in modulating GI function and, consequently, contribute to the manifestation of these disorders [3,4,5].

Despite being functional in nature without identifiable structural or biochemical anomalies, FGIDs pose significant clinical concerns. They are not uncommon in the pediatric population, with many children worldwide experiencing symptoms associated with these disorders [6]. These symptoms often remain refractory to standard treatments. The psychosocial consequences of FGIDs—from anxiety to familial stress—further emphasize the need for a comprehensive understanding of their pathophysiology [7].

FGIDs can impact both the immediate and long-term health and well-being of infants [8,9,10]. In the short run, these effects may manifest as an increased risk of colic, infections, or wheezing [11,12,13,14]. Long-term implications may include a heightened likelihood of developing allergies, asthma, or other immune-related conditions [15,16]. While FGIDs are often studied in isolation, many infants present with overlapping symptoms of multiple FGIDs [12,17].

If microbial dysbiosis plays a role in the development of FGIDs, it raises an intriguing question: can we manage or reverse FGIDs by modulating the gut microbiota? Potential therapeutic approaches may include probiotics (beneficial live microorganisms), prebiotics (nondigestible compounds that stimulate the growth or activity of beneficial gut bacteria), and dietary interventions designed to promote a healthy gut microbial profile [18,19,20,21]. Such microbiota-targeted strategies have shown promise in certain gastrointestinal disorders and could potentially revolutionize FGID management [22,23,24].

However, transitioning from hypotheses to therapeutic application presents numerous challenges. While preliminary studies suggest a correlation between altered gut microbial profiles and FGIDs in children, further research is essential to fully understand this complex relationship.

This study was conducted to investigate the interplay between the gut microbiota and FGIDs in infants. Novel insights into this field were derived from data collection and analysis. It is hoped that these findings will provide clinicians and researchers with a deeper understanding and facilitate the development of personalized therapeutic strategies.

The aim of the study was to analyze the composition of the gut microbiota in infants with functional gastrointestinal disorders (infantile colic, functional constipation, gastroesophageal reflux, and functional diarrhea) based on age, environmental factors, and clinical manifestations.

## 2. Materials and Methods

We performed a prospective longitudinal study on 134 infants aged between 1 month and 12 months during April–September 2024, divided into 2 groups: group I (*n* = 82) included infants with FGIDs, divided into 4 subgroups: group Ia with infantile colic (*n* = 23), group Ib with functional constipation (*n* = 21), group Ic with gastroesophageal reflux (*n* = 20), and group Id with functional diarrhea (*n* = 18); group II consisted of 52 infants without functional gastrointestinal disorders (non-FGIDs). Data on the demographic characteristics were gathered. Written informed consent was obtained from the legal representatives for participation in the study and for the processing of personal data. The study was conducted in accordance with the ethical principles of the Declaration of Helsinki and was approved by the Institutional Review Board of the Clinical Emergency Hospital of Constanta (no. 14472/15.03.2024).

This study involved infants aged 1 to 12 months who underwent a consultation with a pediatric gastroenterologist from the Department of Pediatrics at the Clinical Emergency Hospital of Constanta, during which information regarding the infants’ medical history and gastrointestinal symptoms was gathered. The Rome IV criteria [8] were used to characterize functional gastrointestinal disorders in the studied groups.

The method used for culturing and analyzing dysbiosis involves a bacteriological examination of feces. This process typically includes the following:

1. Sample Collection: Fecal samples are collected during clinical evaluations and delivered to the laboratory within two hours to preserve their integrity.
2. Culturing Techniques: The samples are cultured on specific growth media designed to identify various bacterial groups, such as the following:
  - Putrefactive bacteria (*Escherichia coli*, *Proteus* species, *Klebsiella* species, *Enterobacter* species, *Hafnia alveii*, *Serratia* species, *Providencia* species, *Morganella morganii*, *Kluyvera* species, *Citrobacter* species, *Pseudomonas* species, and *Clostridium* species);
  - Acidifying flora (*Bacteroides* species, *Bifidobacterium* species, *Lactobacillus* species, and *Enterococcus* species);
  - Fungal strains (*Candida albicans*, *Candida* species, and *Geotrichum* species).
  (i) Fecal Sample Collection and Preservation: Fecal samples were collected from infants and immediately stored in anaerobic transport media at 4 °C to prevent bacterial overgrowth and preserve microbial diversity.
  (ii) Selective Media and Culturing Conditions: To isolate specific bacterial groups, we used selective and differential agar plates, including the following:
  - MacConkey agar for Gram-negative bacteria;
  - Chromogenic agar for specific species such as *Escherichia coli* and *Klebsiella*;
  - Bifidobacterium agar for anaerobic bacteria like *Bifidobacterium* species;
  - MRS (de Man, Rogosa, and Sharpe) agar for *Lactobacillus* species;
  - Reinforced Clostridial Medium (RCM) for *Clostridium* species.
  (iii) Incubation: Plates were incubated under appropriate conditions (e.g., anaerobic atmosphere for anaerobes like *Bacteroides* and *Bifidobacterium* or aerobic conditions for other bacterial species) for 24–48 h at 37 °C with subsequent colony enumeration.
  (iv) Biochemical Identification: Colonies from selective media were subjected to biochemical tests to identify and confirm the bacterial species. VITEK systems were used for more precise species identification in certain cases.
  (v) Quantification: The number of CFUs (colony-forming units) per gram of feces was determined for each bacterial group. This involved counting the colonies on each agar plate and calculating the microbial load for each identified species.
  (vi) Fungal Culture: For fungal species identification (e.g., *Candida* and *Geotrichum*), Sabouraud dextrose agar was used, and the cultures were incubated under specific conditions to promote fungal growth.
3. Quantitative and Qualitative Analysis: The bacterial colonies were counted to determine their abundance (colony-forming units, CFUs) and classified based on morphology, biochemical properties, and other specific tests.
4. Flora Index Calculation: A “flora index” was calculated to express the severity of dysbiosis [25], with mild (index 1–5), intermediate (index 6–12), and pronounced (index > 12).

This bacteriological approach allows researchers to characterize the composition and balance of the intestinal microbiota, which is central to evaluating dysbiosis.

Inclusion criteria for the study were represented by an age of 1–12 months, with functional gastrointestinal disorders assessed with laboratory investigations, and without signs and symptoms of acute illness or chronic diseases (genetic diseases, hepatic, renal, metabolic, allergic, cardiac, etc.). The exclusion criteria were represented by an age over 1-year, acute gastrointestinal pathology, symptoms of acute illness or other chronic conditions, and probiotic, prebiotic, and antibiotic treatment that could have influenced the current analysis.

The statistical analysis was performed by using the method of variation statistics with the program Microsoft Excel Version 2312. In the study, descriptive statistics were used to summarize the demographic characteristics of participants. The reliability of the data disparity in the comparative groups was evaluated using the t-test. The significant level of *p*-values was established at *p* < 0.05. Statistical methods included descriptive statistics for group characteristics (mean, standard deviation), the *t*-test, and the flora index to classify dysbiosis severity.

## 3. Results

In the FGID group, 57.3% cases were male and 42.7% female. Except for the type of feeding, no significant differences were observed in the gestational age, mode of delivery, or birth weight (Table 1).

Specific characteristics were identified in the composition of the gut microbiota in the infantile colic group (Table 2), who presented a significant decrease in acidifying germs such as *Lactobacillus* species (*p* = 0.014), *Bifidobacterium* species (*p* = 0.031), *Bacteroides* species (*p* = 0.038), and *Enterococcus* species (*p* = 0.002). Putrefactive germs presented high levels of Proteobacteria represented by *Escherichia coli* (*p* = 0.021) and *Klebsiella* species (*p* = 0.028) (Table 2). The flora index was suggestive for intermediate dysbiosis, with a mean value of 6.0.

Infants with functional constipation (Table 2) presented significantly lower levels of acidifying bacteria *Lactobacillus* species (*p* = 0.028), *Enterococcus* species (*p* = 0.036), and *Bacteroides* species (*p* = 0.024), with increased levels of those in the Enterobacteriaceae family, namely *Escherichia coli* (*p* = 0.019) and *Klebsiella* species (*p* = 0.038). The flora index was suggestive for intermediate dysbiosis (mean value 6.2).

In the group of infants with gastroesophageal reflux (Table 2), decreased values of *Lactobacillus* species (*p* = 0.023) and *Bacteroides* species (*p* = 0.031) were observed, and high levels of *Enterococcus* species (*p* = 0.035), *Escherichia coli* (*p* = 0.044), and *Klebsiella* species (*p* = 0.012) were observed. The flora index was suggestive of intermediate dysbiosis (mean value 6.0).

Functional diarrhea (Table 2) was associated with high levels of *Escherichia coli* (*p* = 0.035), *Enterobacter* species (*p* = 0.042), and *Klebsiella* species (*p* = 0.026), as well as a lower abundance of *Bacteroides* species (*p* = 0.046), *Lactobacillus* species (*p* = 0.031), and *Enterococcus* species (*p* = 0.039), with the flora index suggestive of intermediate dysbiosis (mean value 6.5).

Microbial levels of *Proteus* species, *Hafnia alveii, Citrobacter* species, *Serratia* species, *Providencia* species, *Kluyvera* species, *Pseudomonas* species, *Clostridium* species, and *Morganella morganii* presented normal values in both FGID and non-FGID groups (Table 2). Fungal species *Candida albicans* and *Geotrichum* species presented normal values in both FGID and non-FGID groups (Table 2).

Infants who received breastfeeding presented a decreased risk for intestinal dysbiosis in FGIDs (25.6%), while infants who received infant formula had a high risk of developing dysbiosis (58.9%), with a lower abundance of acidifying flora (*Lactobacillus* species, *Bifidobacterium* species, *Bacteroides* species) and increased levels of putrefactive bacteria (*Escherichia coli* and *Klebsiella* species). It was observed that infants born by *C*-section presented an increased risk of intestinal dysbiosis (81.7% cases, *n* = 67).

In the FGID group, a flora index suggestive of intermediate dysbiosis (flora index between 6 and 12) in 63.4% of cases (*n* = 52), mild dysbiosis (flora index between 1 and 5) in 25.6% of cases (*n* = 21), and pronounced dysbiosis (flora index > 12) in 11% of cases (*n* = 9 cases) was observed, with elevated pH values of feces in 79.2% of cases (*n* = 65). An elevation in the concentration of proteolytic bacteria was noted alongside alkaline fecal pH values.

## 4. Discussion

Infants with functional gastrointestinal disorders presented an imbalance of intestinal microbiota, which was characterized by a significant decrease in the main representatives of Lactobacillales-order microflora represented by *Lactobacillus* and *Enterococcus* and low levels of *Bacteroidetes* phylum represented by *Bacteroides* species. A high abundance of proteolytic microorganisms from the Enterobacteriaceae family such as *Klebsiella* species and *Escherichia coli* was observed. Saeed et al. [26] reported a high abundance of Proteobacteria and a lower abundance of *Lactobacillus* and *Bifidobacterium* in infant colic, as well as high levels of *Lactobacillus* and low levels of *Bifidobacterium* in functional abdominal pain.

*Clostridium difficile* causes inflammation and damage to the intestinal lining, which can lead to increased mucus production [27]. Increased mucus production is a response to inflammation, helping to protect the intestinal lining from further damage. *Bacteroides* species produce enzymes that can determine inflammation in the intestine [28]. However, in cases of severe or persistent bacterial dysbiosis, excess mucus production can lead to symptoms such as abdominal pain and modified stools with the presence of mucus [29].

It was suggested that preterm newborns have a reduced microbial diversity, with the presence of a potentially pathogenic composition, with initial colonization including *Enterobacteria*, lactobacilli, and *Escherichia coli* replaced by *Bifidobacterium*, *Bacteroides*, and *Clostridium* [30]. Studies reported a decrease in *Bifidobacterium* spp. and higher levels of Ruminococcaceae, suggesting the transition from a bifidobacteria-rich gut composition to a diverse community, with butyrate and propionate producing bacteria [31]. Di Profio et al. [31] showed that formula-fed infants present a gut microbiota characterized by a lower abundance of *Bifidobacterium* species and increased levels of *Clostridium* species and the Enterobacteriaceae family. The presence of Enterobacteriaceae enrichment indicates a compromised composition of gut microbiota in infants delivered via *C*-section, demonstrating the efficacy of probiotic treatment with particular strains of *Bifidobacterium* and *Lactobacillus* after birth in infants [32].

Infant colic is characterized by an increased abundance of Proteobacteria and a decreased presence of the genera *Lactobacillus* and *Bifidobacterium*, resulting in a reduced variety of gut bacteria [33]. Our findings confirmed a significant decrease in *Lactobacillus* species, *Bifidobacterium* species, *Bacteroides* species, and *Enterococcus* species, while putrefactive flora presented high concentrations of *Escherichia coli* and *Klebsiella* species. However, Savino et al. discovered that there were no discernible disparities in the levels of *Bifidobacteria* in colicky infants [34]. In contrast, another study reported higher levels of *Lactobacillus* [34]. The phylum Bacteroidetes was identified to be predominant in infant colic in several studies, with significantly lower levels during their first two months of life [35,36]. The mechanism by which probiotics improve newborn colic has not been definitively established, although it is believed that they may exert their effects by altering the function of the intrinsic sensory neurons in the colon, so enhancing gut motility [37,38]. Furthermore, they exert beneficial effects on both function and visceral pain [2,39].

Infants with functional constipation presented decreased concentrations of *Lactobacillus* species and *Enterococcus* species and increased levels of *Escherichia coli* and *Klebsiella* species. Other studies reported a reduced abundance of *Lactobacillus* in infant constipation [40].

A lower abundance of *Lactobacillus* and *Bacteroides* was observed in infants with gastroesophageal reflux, while *Enterococcus* species, *Escherichia coli*, and *Klebsiella* species were significantly higher. Korpela et al. [41] reported lower levels of *Lactobacillus* in infants with diarrhea and gastroesophageal reflux. Our findings showed the association between functional diarrhea and a high abundance of the Enterobacteriaceae family such as *Escherichia coli* and *Klebsiella* species, as well as decreased values of *Lactobacillus* and *Bacteroides* species.

Targeting the gut microbiota for therapeutic intervention in FGIDs is a subject of considerable interest. The current therapeutic approach for microbiota-directed therapies in FGIDs involve the modulation of the gut microbiota by probiotics, prebiotics, synbiotics, fecal microbiota transplantation (FMT), and dietary modifications for the modulation of gut microbiota.

The gut microbiota and its metabolites may be crucial factors in the pathophysiology of FGIDs. The clarification of the production steps may provide new treatment possibilities for the release and utilization of these compounds. This knowledge would provide improved therapy strategies, enabling the precise restoration of disrupted physiological functioning in FGIDs. Novel therapeutic interventions targeting the gut microbiota may include the introduction of specific microbes capable of reinstating normal gut functions by synthesizing essential microbiota-derived molecules, exemplified by genetically engineered *Bacteroides thetaiotaomicron* that generates tryptamine or the inhibition of specific microbial processes via inhibitors targeting enzymatic pathways [4].

### Limitations

The study is constrained by considerable limitations (Table 3). The study group included a relatively small number of participants, although it is precisely defined. Initially, it did not analyze stool samples prior to the onset of FGIDs, hence hindering the identification of a temporal link. The composition of gut microbiota may vary considerably among individuals. In this specific age group, the gut microbiota has continuous development. The description of potential influencing factors on gut microbiota was not exhaustive. The group is homogeneous; thus, the results can only refer to this population, and conclusions cannot be drawn across the entire population. The sensitivity of statistical analysis and culture-based CFU quantification is limited by the small sample size and microbial variability, influenced by individual factors. Specificity is also influenced by culture-dependent biases. Future research should integrate larger datasets and metagenomic sequencing to improve accuracy. Additionally, metagenomic sequencing should be integrated to extend the study beyond gut microbiota composition, providing deeper insights into the functional role of the gut microbiome in FGIDs.

## 5. Conclusions

Intestinal microbiota in infants with gastrointestinal disorders is characterized by a lower abundance of acidifying species such as *Lactobacillus*, *Bifidobacterium*, and *Enterococcus* and high concentrations of proteolytic microorganisms from the Enterobacteriaceae family such as *Klebsiella* species and *Escherichia coli*, which could be one of the possible trigger factors for the occurrence of functional gastrointestinal disorders. In this study, we investigated the interplay between the gut microbiota and functional gastrointestinal disorders (FGIDs) in infants, aiming to shed light on this complex relationship. Our findings provide a novel perspective in this growing field, offering valuable insights into the role of gut microbiota in the development and progression of FGIDs. By enhancing our understanding of these mechanisms, we hope to contribute to the advancement of personalized therapeutic strategies for managing FGIDs in infants.

Cesarean delivery represents a risk factor for the occurrence of gut dysbiosis in infants compared with vaginal delivery due to deprivation of commensal bacteria from the maternal vaginal area. The type of feeding in the first year of life has an important role in the development of the gut microbiota and influences the concentration of beneficial bacteria.

## Figures and Tables

**Table 1:** Baseline characteristics of FGID group and non-FGID group.

Characteristic	FGID Group (*n* = 82)	Non-FGID Group (*n* = 52)
Male, N (%)	47 (57.3)	27 (51.9)
Female, N (%)	35 (42.7)	25 (48.1)
Age months, mean (SD)	5.37 (1.2)	6.43 (1.4)
Mode of delivery		
Vaginal delivery, N (%)	30 (36.6)	23 (44.2)
Cesarean section, N (%)	52 (63.4)	29 (55.8)
Type of feeding		
Breastfeeding, N (%)	21 (25.6)	28 (53.8)
Formula feeding, N (%)	49 (59.8)	16 (30.8)
Mixed feeding (infant formula and breastfeeding), N (%)	12 (14.6)	8 (15.4)
Gestational age		-
Under 37 weeks	15 (18.3)	9 (17.3)
37–42 weeks	62 (75.6)	41 (78.9)
Over 42 weeks	5 (6.09)	2 (3.8)
Birth weight		
Under 2500 g	11 (13.4)	5 (9.6)
2500–4000 g	69 (84.1)	43 (82.7)
Over 4000 g	2 (2.5)	4 (7.7)

**Table 2:** The composition of intestinal microbiota in infants with FGIDs.

Microorganisms (CFU/g)	Group Ia (Infantile Colic, *n* = 23)	Group Ib (Functional Constipation, *n* = 21)	Group Ic (Gastroesophageal Reflux, *n* = 20)	Group Id (Functional Diarrhea, *n* = 18)	Group II (Non-FGIDs, *n* = 52)
*Escherichia coli* (×10^7^)	9.81 ± 0.62 (*p* = 0.021)	10.04 ± 1.02 (*p* = 0.019)	9.84 ± 0.73 (*p* = 0.044)	9.96 ± 0.68 (*p* = 0.035)	1.2 ± 0.41
*Proteus* species (×10^4^)	0.81 ± 0.08 (*p* = 0.722)	0.89 ± 0.03 (*p* = 0.842)	0.86 ± 0.06 (*p* = 0.125)	0.82 ± 0.04 (*p* = 0.613)	0.94 ± 0.03
*Klebsiella* species *(*×10^4^)	2.06 ± 1.04 (*p* = 0.028)	6.86 ± 1.32 (*p* = 0.038)	2.76 ± 1.22 (*p* = 0.012)	4.72 ± 1.08 (*p* = 0.026)	0.87 ± 0.06
*Enterobacter* species (×10^4^)	0.89 ± 0.04 (*p* = 0.621)	0.84 ± 0.14 (*p* = 0.613)	0.81 ± 0.12 (*p* = 0.380)	3.11 ± 0.86 (*p* = 0.042)	0.81 ± 0.08
*Hafnia alveii* (×10^4^)	0.93 ± 0.05 (*p* = 0.726)	0.91 ± 0.04 (*p* = 0.632)	0.93 ± 0.05 (*p* = 0.913)	0.91 ± 0.02 (*p* = 0.515)	0.89 ± 0.05
*Serratia* species (×10^4^)	0.96 ± 0.03 (*p* = 0.724)	0.93 ± 0.05 (*p* = 0.813)	0.86 ± 0.11 (*p* = 0.558)	0.88 ± 0.07 (*p* = 0.656)	0.91 ± 0.02
*Providencia* species (×10^4^)	0.89 ± 0.06 (*p* = 0.851)	0.90 ± 0.03 (*p* = 0.747)	0.87 ± 0.09 (*p* = 0.766)	0.87 ± 0.11 (*p* = 0.632)	0.86 ± 0.03
*Morganella morganii* (×10^4^)	0.87 ± 0.04 (*p* = 0.788)	0.89 ± 0.08 (*p* = 0.932)	0.91 ± 0.05 (*p* = 0.308)	0.88 ± 0.11 (*p* = 0.521)	0.87 ± 0.05
*Kluyvera* species (×10^4^)	0.90 ± 0.05 (*p* = 0.964)	0.94 ± 0.03 (*p* = 0.614)	0.85 ± 0.13 (*p* = 0.601)	0.92 ± 0.06 (*p* = 0.813)	0.92 ± 0.04
*Citrobacter* species (×10^4^)	0.82 ± 0.11 (*p* = 0.821)	0.84 ± 0.12 (*p* = 0.775)	0.88 ± 0.09 (*p* = 0.142)	0.95 ± 0.03 (*p* = 0.543)	0.93 ± 0.05
*Pseudomonas* species (×10^4^)	0.91 ± 0.04 (*p* = 0.910)	0.89 ± 0.10 (*p* = 0.711)	0.89 ± 0.08 (*p* = 0.114)	0.91 ± 0.07 (*p* = 0.562)	0.91 ± 0.03
*Clostridium* species (×10^5^)	0.96 ± 0.03 (*p* = 0.714)	0.91 ± 0.06 (*p* = 0.819)	0.96 ± 0.03 (*p* = 0.637)	0.94 ± 0.03 (*p* = 0.628)	0.90 ± 0.04
*Bacteroides* species (×10^8^)	0.82 ± 0.08 (*p* = 0.038)	0.87 ± 0.05 (*p* = 0.024)	0.74 ± 0.05 (*p* = 0.031)	0.76 ± 0.04 (*p* = 0.046)	1.43 ± 0.32
*Bifidobacterium* species (×10^8^)	0.85 ± 0.06 (*p* = 0.031)	1.46 ± 0.35 (*p* = 0.736)	1.18 ± 0.16 (*p* = 0.681)	0.95 ± 0.03 (*p* = 0.814)	1.36 ± 0.21
*Lactobacillus* species (×10^5^)	0.72 ± 0.09 (*p* = 0.014)	0.73 ± 0.12 (*p* = 0.028)	0.78 ± 0.06 (*p* = 0.023)	0.71 ± 0.4 (*p* = 0.031)	1.74 ± 0.23
*Enterococcus* species (×10^6^)	0.76 ± 0.06 (*p* = 0.002)	0.79 ± 0.19 (*p* = 0.036)	11.03 ± 1.14 (*p* = 0.035)	0,81 ± 0.05 (*p* = 0.039)	1.81 ± 0.28
*Candida albicans* (×10^3^)	0.94 ± 0.04 (*p* = 0.931)	0.86 ± 0.04 (*p* = 0.530)	0.92 ± 0.05 (*p* = 0.561)	0.95 ± 0.03 (*p* = 0.986)	0.96 ± 0.02
*Geotrichum* species (×10^3^)	0.95 ± 0.02 (*p* = 0.645)	0.96 ± 0.02 (*p* = 0.862)	0.89 ± 0.03 (*p* = 0.611)	0.89 ± 0.06 (*p* = 0.935)	0.90 ± 0.04

**Table 3:** Limitations of the study.

Limitation	Description
Small Sample Size	The relatively small number of participants constrains the generalizability of the findings. Future studies with larger cohorts are needed to confirm these results.
Lack of Pre-FGID Stool Samples	Stool samples were not collected before the onset of FGIDs, limiting the capacity to establish a temporal relationship between gut microbiota changes and the development of FGIDs.
Inter-Individual Variability	Gut microbiota composition varies among individuals, and while efforts were made to control for confounding factors, genetic and environmental influences were not exhaustively analyzed.
Developmental Changes in Gut Microbiota	The dynamic characteristics of the gut microbiota during infancy means that findings may not be fully representative of long-term microbial patterns.
Homogeneous Population	The study population was relatively homogeneous, which may limit the applicability of findings to wider populations.
Culture-Based Methodology Bias	The use of traditional culture-based techniques may result in biases, as certain anaerobic and less common bacterial species are difficult to culture, thus causing underrepresentation.
Limited Analysis of External Influencing Factors	Factors such as maternal diet were not comprehensively evaluated, which could have influenced gut microbiota composition.
Statistical Sensitivity Constraints	Given the sample size, the statistical power to detect subtle differences in microbiota composition may be limited. Larger datasets would improve sensitivity and accuracy.
Future Research Directions	Future research should integrate high-throughput sequencing, larger cohorts, and a broader range of influencing factors to enhance the understanding of gut microbiota dynamics in FGIDs.

## Data Availability

The data presented in this study are available upon request from the corresponding author due to ethical concerns.
